# Enhanced Milieu Teaching with Phonological Emphasis: A Pilot Telepractice Parent Training Study for Toddlers with Clefts

**DOI:** 10.3390/children8090736

**Published:** 2021-08-26

**Authors:** Jennifer Philp, Paige K. Ellis, Nancy J. Scherer, Kari M. Lien

**Affiliations:** 1 Barrow Cleft and Craniofacial Center, 124 W Thomas Rd 3rd Floor, Phoenix, AZ 85013, USA; jphilp714@gmail.com (J.P.); kari.lien@dignityhealth.org (K.M.L.); 2 College of Health Solutions, Arizona State University, Tempe, AZ 85281, USA; pellis5@asu.edu

**Keywords:** cleft palate, early intervention, speech and language development, telepractice

## Abstract

Objective: the purpose of this study was to evaluate the effects of training caregivers to use intervention strategies from the Enhanced Milieu Teaching with Phonological Emphasis (EMT + PE) program, delivered via telepractice, and to examine the effects on child speech and language outcomes for children with repaired cleft lip +/− palate (CL/P). Design: A multiple baseline within subject design across parent behaviors was replicated across three participating dyads. A pre–post intervention comparison was provided with a non-cleft twin. Participants: Three mother-child dyads participated in this study. Children ranged in age from 21 to 27 months at the beginning of the study and all had a diagnosis of CL/P. A noncleft twin without CL/P was assessed pre- and post-intervention to provide a normative comparison. Results: Parents demonstrated a positive intervention effect by substantially increasing their use of EMT + PE intervention strategies during telepractice intervention sessions (Tau 0.675 to 1.1333). Following the conclusion of intervention, parents were able to maintain their use of strategies once direct coaching had been discontinued. Children demonstrated increased talking rate, improved speech production and expanded expressive vocabulary measures over the course of intervention. Speech and language development of a child without cleft palate was provided as a comparison. Conclusions: Parents were trained through telepractice to effectively deliver EMT + PE speech and language facilitation strategies that resulted in increased language and speech outcomes for their children with CL/P.

## 1. Introduction

Cleft lip +/− cleft palate (CL/P) is a congenital craniofacial condition that affects approximately 1 out of every 750 live births in the United States each year [1]. Children born with CL/P are at risk for delayed speech sound development and early expressive language development that persists for some children into the preschool and early school-aged years [2,3]. A recent meta-analysis of early speech and language development indicated that, as a group, children with nonsyndromic CL/P demonstrate reduced articulation and receptive and expressive language skills when compared to children without clefts through eight years of age [4]. The meta-analysis indicated that early language and speech delays persist into the early elementary years which could impact school achievement, making early intervention services crucial to improve outcomes in this clinical population.

A 2013 systematic review of speech and language interventions for individuals with cleft palate reviewed 17 intervention studies for children and adults [5]. The review compared linguistic/phonological approaches with motor-phonetic approaches. While all of the studies reported significant findings, no one intervention approach was more efficacious based on the limited studies conducted. Additionally, many of the studies were found to lack methodological rigor and/or were not adequately powered to provide reliable evidence of treatment effects. However, a recent small randomized control intervention study compared these two approaches for four to twelve year old children and found that while both approaches improved consonant inventory, the linguistic/phonological approach was more effective than the motor-phonetic approach for improving speech outcomes [6]. While these approaches have been tested on children over four years of age, children under four often require adaptation of these approaches to naturalistic conditions.

There are several evidence-based early intervention approaches, such as a parent-implemented focused stimulation and enhanced milieu teaching (EMT) that have been used with young children with CL/P [7,8,9,10]. Both models have shown increased sound inventories, increased speech accuracy, and decreased compensatory articulation errors in children with cleft palate following parent training. However, an adaptation of EMT, Enhanced Milieu Teaching with Phonological Emphasis (EMT + PE), has provided evidence-based data to support vocabulary use and expand sound inventories and accuracy for young children with nonsyndromic CL/P [8,10,11]. EMT + PE is a naturalistic intervention that provides support for developmental change by focusing on both vocabulary and speech targets simultaneously. In a study of young children with nonsyndromic CL/P, clinician implemented EMT + PE was demonstrated to significantly improve receptive language, expressive vocabulary, and percent consonants correct (PCC) as compared to a group of children with CL/P who received typical community-based services [8]. A subsequent study in Brazil compared clinician implemented EMT + PE, which included a parent training component, to a business-as-usual group and found significant increases in vocabulary and speech sound acquisition in the intervention group [12]. Additionally, the children whose parents received training continued to make speech and language gains three months following the intervention. Other studies of children without cleft palate but with speech and language delays have shown that parents can learn to implement the EMT strategies reliably to support speech and language acquisition [13,14,15]. In a 2011 meta-analysis evaluating 18 group-design intervention studies, it was concluded that parents trained in naturalistic techniques did have a positive impact on the child’s communication skills [13,16].

Telepractice delivery of parent training in early intervention has been used for a number of years but until recently, few studies have compared its effectiveness with traditional face-to-face interaction [17]. A recent systematic review of parent-implemented early intervention via telepractice in children without cleft palate but with disabilities found that for most intervention components, telepractice and traditional face to face delivery models resulted in significant gains in use of trained intervention strategies and improved child outcomes [18]. Parent training has typically occurred in face-to-face sessions where the clinician demonstrates strategies with the child and then gives the parent the opportunity to use the strategy while being coached by the clinician in a teach–model–coach–review model (TMCR) [15]. However, delivering this model through telepractice alters the “model” and “coach” portions of the training. The modeling that the clinician provided with the parent’s child is now replaced with video examples of the clinician modeling the strategy with another child or watching video examples in a video library. Some researchers have provided annotated or written coaching feedback to parent submitted videos [19]. Others have used a hybrid model of synchronous and asynchronous training in which modules are available online for parents to review and face-to-face sessions are used for feedback and direct observation of parents’ implementation [20]. While the focus of these studies has been on training parents to use language intervention strategies, there has been little consideration of early intervention that focuses on training parents to facilitate language and speech production simultaneously.

Young children with CL/P present with speech sound production delays and resulting vocabulary delays due to their early structural deficits [21,22,23]. These delays persist for some children resulting in delayed acquisition of phonology and use of speech sound errors that are unique to children with cleft palate. These speech characteristics include compensatory articulation errors (e.g., glottal stops, pharyngeal fricatives), nasal substitutions related to learned behaviors, obligatory nasalization and nasal emission related to structural causes. While some of these cleft related speech errors may be related to velopharyngeal dysfunction, some may persist following palate repair as a learned pattern. Telepractice and traditional face-to-face service delivery were found to be equivalent for improving speech sound disorders in noncleft school-aged children with speech sound disorders [24]. Sweeney, et al. (2020) conducted a randomized control trial of a parent led therapist supervised articulation therapy (PLAT) for 2;9–7;5 year old children with cleft palate [25]. They found that speech articulation improved equally when delivered by parents, coached by SLPs via telepractice, and traditional intervention from an SLP. This intervention was extended to clinical settings in a small pilot study and the results were replicated in a larger study suggesting that parents coached via telepractice could facilitate speech gains similar to those obtained from traditional therapy with an SLP. These findings suggest that telepractice can be a viable delivery model for articulation therapy; however, telepractice effectiveness has not been studied with children less than three years of age.

The purpose of this study was to evaluate the effects of parent training in the Enhanced Milieu Teaching with Phonological Emphasis Intervention Program, using telepractice, on parent strategy use and child speech and language outcomes for children with repaired cleft lip and/or palate and a comparison twin without cleft palate.

Research Questions

1. Does parent use of EMT + PE strategies, including (a) matched turns and environmental arrangement, (b) modeling and expansions, and (c) prompting and speech recasting increase with parent-training on these specific strategies during intervention and maintain following the intervention?
2. Do the children’s consonant inventory, percent of consonants correct (PCC), word structure match (WSM), whole word accuracy (ACM) and expressive language use result in significant effect size differences between pre-intervention to post-intervention? And how do these changes compare with the performance of a noncleft twin who did not participate in the intervention?

## 2. Materials and Methods

The study was approved by the Arizona State University Institutional Review Board (IRB). Four child-parent dyads were recruited for this study. Three dyads met inclusion criteria and were selected for participation in the intervention study [26,27,28]. Participants were recruited through a local cleft-craniofacial team, social media support groups, and through local speech and language centers. One twin without cleft palate was also included for comparison at pre- and post-intervention.

Three child participants who received intervention had a cleft lip/alveolus or cleft lip and palate participated in the study, along with one child without cleft palate who was a twin of a participant with a cleft. All children met the following criteria: (1) between 20 and 30 months of age at pre-intervention, (2) for children with clefts involving the palate, underwent primary palate repair by 12 months of age, (3) no prior history of speech therapy services, (4) demonstrated cognitive performance within the normal range as indicated by a cognitive composite score of 80 or above on the Bayley Scales of Infant and Toddler Development-III, (5) demonstrated joint attention with a caregiver during play as demonstrated by receiving a score of “1” on Expressive Communication Item #20 on the Preschool-Language Scales, Fifth Edition (PLS-5). Participants were excluded from the study if their home language was a language other than English. The twin without cleft had no reported speech and language concerns by the parent. Pre-intervention assessment of this child confirmed typical development. Participant demographics at pre-intervention is shown in Table 1.

### 2.1. Study Design

This study was designed as a multiple baseline across parent behaviors research design. Intervention was replicated across three participating dyads [28]. Additionally, a pretest-posttest comparison of child speech and expressive language measures was obtained for the three children engaged in the intervention and a twin who did not receive the intervention. Prior to the introduction of any treatment phase, a stable baseline for each treatment phase was established and judged to be ready for intervention by a masked visual analyst (MVA) [29]. Once a stable baseline had been indicated by the MVA for each individual parent, they received training on the next phase of intervention strategies. The MVA also indicated when each parent was ready to proceed to the next phase of treatment strategies once parents had reliably increased their frequency of strategy use by at least 10%. The number of sessions from the beginning of baseline to the end of maintenance ranged from 23 to 27 sessions.

#### 2.1.1. Pre–Post Intervention Assessment

A comprehensive assessment battery of speech, language and cognition was administered at pre-intervention and post-intervention. The sessions were video recorded at pre intervention in the Arizona State University Craniofacial Laboratory and in post intervention using Zoom [30] due to the COVID-19 shut down. A LENA audio recorder [31] was worn by the child at both time points. The cognitive scale from the Bayley Skills of Infant Development-III [32], was individually administered at pre-intervention by a licensed SLP trained to administer this cognitive test. The child’s receptive and expressive language abilities were assessed using, (a) the McArthur Bates Communicative Development Inventory [33]; (b) 15-min language samples with parent-child interaction collected to obtain number of different words (NDW), total number of words (TNW), mean length of utterance in morphemes (MLUm), words per minute (WPM), and number of vocalizations; and (c) the Preschool Language Scales—Fifth Edition (PLS-5) [34]. The Profiles of Early Expressive Phonological Skills (PEEPS) was administered to obtain a consonant inventory, total percent consonants correct (PCC) score, percent consonants correct for stops (PCC Stops), word structure match (WSM) and whole word accuracy match (ACM) score [35]. Speech intelligibility was measured using the intelligibility in context scale, which is a parent-report social validity measure (ICS) [36].

The PLS-5 was attempted at post-intervention through teleconferencing; however, the administration was not considered reliable. The profiles of early expressive phonological skills (PEEPS) was virtually administered to each individual child using a document camera to present each assessment item. All language samples between parent and child were collect through Zoom and transcribed by trained student clinicians using Systematic Analysis of Language Transcripts (SALT) software [37]. Samples were coded for each individual language facilitation strategy and analyzed for the measures listed above. Speech accuracy results obtained from the PEEPS were transcribed by a trained student clinician who was not the interventionist and re-transcribed for reliability by a second student clinician. Reliability was evaluated by a licensed SLP and consensus was determined for all disagreements.

Target Word Selection: Following assessment, speech and language results were analyzed, and individualized goals were identified for each child. Vocabulary and speech sound targets were addressed together. Target words consisted of early developing low pressure sounds such as /h, m, n, w, j/ and high pressure stop consonants including /p, b, t, d, k, g/. A set of 10 probe picture cards with target words were provided to each parent and updated as the children acquired consistent production of target sounds in words over three sessions. Parents were encouraged to have their child name the cards while wearing the LENA and clinicians transcribed the probes following intervention sessions to track speech accuracy.

Intervention Activities: Activities were selected with parent consultation to embed toys and activities that contained target sounds and words. Three to four activities were discussed prior to each intervention session so that parents could have play sets ready for the session. Parents were asked to complete a toy list for items they had at home prior to the beginning of intervention, and these were supplemented by the project so that all families had the same range of toys to use in the intervention.

#### 2.1.2. Baseline Phase

During the baseline phase, sessions were conducted with a standard set of toys and activities selected with the parent to engage with the child. Data were collected on independent use of parent strategies prior to the introduction of intervention over a minimum of five 15-min sessions. The baseline phase lasted between one to two weeks to allow time for sufficient data to be collected on the parent use of strategies and child responsiveness. All collected data points were plotted and sent to the MVA. The MVA determined participant eligibility to advance from the baseline to the intervention phase when a stable baseline was established [26,27,28].

#### 2.1.3. Intervention Phase

The intervention phase included parent training and subsequent coached intervention sessions in three phases. Parent training sessions utilized the teach–model–coach–review (TMCR) method of parent training [13,15]. The TMCR approach was adapted for the telepractice environment [15]. During this approach, parents were trained in specific strategies with Powerpoint presentations that included video demonstration examples and practice activities (teach-model). Parents were then given an opportunity to use the strategy in an activity with their child as the clinician coached them to successfully use the strategies (coach). Finally, strategies and performance were reviewed and discussed at the conclusion of the session (review). In our telepractice adaptation, the *teach* component of the intervention included an in-person overview of the language and speech support strategy by way of a PowerPoint presentation outlining the strategy. Multiple video and written examples of each strategy were used. The *model* component of the intervention included real-life examples of an interventionist demonstrating the strategies with a child. Parents were invited to observe 10–15 min of the new strategies being modeled in-person with their child by a graduate student clinician and a licensed SLP in a therapy room. The *coach* component included parent practice of the strategy use with their child in routine and play activities while receiving feedback and guidance from the interventionist. Parents took over play in the therapy room for 10–15 min while the clinician coached them on their strategy use in play. Each time the parent successfully used the strategies, they were provided with a positive verbal reinforcement. During the online intervention sessions, the coach component was implemented by way of feedback from the clinician throughout the session. Coaching was dispersed throughout the session across 5–7-min increments. The *review* component of the intervention included evaluation and reflection methods for reviewing the session and determining a plan for next steps. The clinician recapped moments of strong strategy implementation and provided suggestions for enhanced implementation, when appropriate. At the conclusion of the intervention session, the clinician and parents discussed the plan for the next session and suggestions for practice in daily routine activities.

Intervention Strategies: EMT + PE intervention strategies included environmental arrangement and matched turns, modeling and expansions, and prompting and speech recasting. *Environmental arrangement* focused on arranging furniture or equipment to create engagement and manipulation of materials to maintain child interest and engagement during activities. *Matched turns* focused on following the child’s lead, allowing the child to choose the play materials from a pre-determined set, playing face-to-face, demonstrating rather than telling the child what to do and commenting on the child’s play rather than questioning. *Modeling* taught the parent to label an object, a change in location, and/or an action in the child’s play. *Expansions* taught the parent to add on to the child’s utterance with a new or different word, and/or turn the child’s utterance into a sentence. *Prompting* taught parents to provide their child with verbal support to encourage target word use and increased language use and complexity through say prompts (i.e., “say dog”), choice questions (i.e., “Do you want the dog or the ball?”), and open questions (i.e., “what are you doing?”). *Speech recasting* trained parents to repeat their child’s incorrectly produced word while using accurate adult articulation, emphasizing the pronunciation of the target sound in the word (i.e., “The dog is running”). Strategies were grouped in training if they supported each other in application. For example, environmental arrangement was used to teach parents how to match child actions and interests in play. Modeling and expansions were taught together based on their frequent use together in natural interaction. More detailed descriptions of the intervention strategies are provided in the Supplementary Materials.

During the intervention phase, a range of 20–24 sessions were conducted. This included three foundational in-person parent-training workshops, during which new strategies were introduced, and 17–21 parent intervention sessions. The total number of sessions varied across participants with rate of parent strategy use during the intervention phase. Intervention sessions were conducted over three 30-min weekly sessions using video teleconferencing. After the introduction of each new parent strategy during the training workshops, intervention sessions were held using TMCR to reinforce parent strategy use until a criterion increase of parent strategy use of at least 10% was achieved for three consecutive sessions.

Parents were coached on strategy use and data were collected for the first five minutes each session. These five-minute samples were later transcribed using the SALT software and coded for parent use of each trained strategy. During five-minute data collection sessions, parents produced approximately 100 utterances and percent of strategy use was calculated for each strategy. The number of instances of target strategy use was added and divided by the number of total parent utterances within the five minute sample to determine percent strategy usage. Additionally, during each intervention session, parents were asked to elicit the speech sound probe words provided to collect data on speech sound development for each child. The five-minute language samples were further analyzed to obtain the identified speech measures during spontaneous speech sound productions for each child. At the end of each session the clinician and parent discussed the toys to have available for the next session and reviewed which speech and language targets to focus on between intervention sessions.

#### 2.1.4. Maintenance Phase

The maintenance phase followed the same procedure as the baseline phase in that data were collected on parent strategy use although parents were not coached on their use of the strategies. Three weekly data collection sessions began two weeks after the conclusion of post-intervention assessment sessions to assess parent use of strategies. A total of four maintenance sessions were completed with each family. The attached supplementals provide a detailed outline of intervention session procedures. At the end of the maintenance phase parents were asked to complete a post intervention survey.

#### 2.1.5. Reliability and Procedural Fidelity

Both intra- and inter-rater reliability was calculated for a minimum of 20% of all language samples that were transcribed and coded for parent strategy during each phase of this study. In phases where 20% of transcriptions were equal to fewer than two transcripts, a minimum of two transcripts were re-coded instead. At least 88% agreement for both intra-rater (88–100%) and inter-rater (88–100%) reliability was achieved for all transcriptions and codes for parent strategies. Similarly, inter-rater reliability was calculated for all phonetic transcriptions of child speech measures and a minimum of 80% agreement (81–100%) was achieved. All disagreements were resolved through consensus coding. A rating of procedural fidelity was obtained for each parent across all phases. This allowed for tracking the percent of successfully implemented strategies as compared to a set criterion (matched turns at 75%, modeling and expansions at 40%, prompting and speech recasting at 35%). The criterion levels originated in the work by Kaiser, et al., (2017) and Scherer, et al., (2020) [7,8]. The procedural fidelity form is provided in Table 2.

### 2.2. Analysis

The effects of parent training were analyzed by examining the data for intervention effects and evaluating effect size magnitude [28]. The MVA inspected whether or not a consistent baseline had been established to move on to intervention. Visual inspection of data for intervention effects was utilized within phases for each participant and across all three participants. Visual inspection of the data included level, trend, variability, immediacy of effect, overlap, and consistency [24]. Additional quantitative analysis was conducted to support findings of our visual inspection. Magnitude of effect was determined using the following calculations commonly utilized in single-case research design. Percentage of nonoverlapping data (PND) was used to allow for descriptive degrees of overlap and the Tau-nonoverlap measure (Tau-U) was used as an additional calculation of effect size. Tau values account for both the level change across phases and positive baseline trend [38].

## 3. Results

The results of the parent training are presented first followed by pre–post speech and language assessment comparisons for the three children with clefts who received the intervention and the child without cleft who did not receive intervention. Our comparison child did provide a developmental noncleft comparison for one of our intervention children, although her mother was trained in the intervention strategies and she likely was exposed to these strategies without engaging directly in the intervention sessions.

### 3.1. Parent Strategy Use over the Course of Intervention

**Figure 1:**
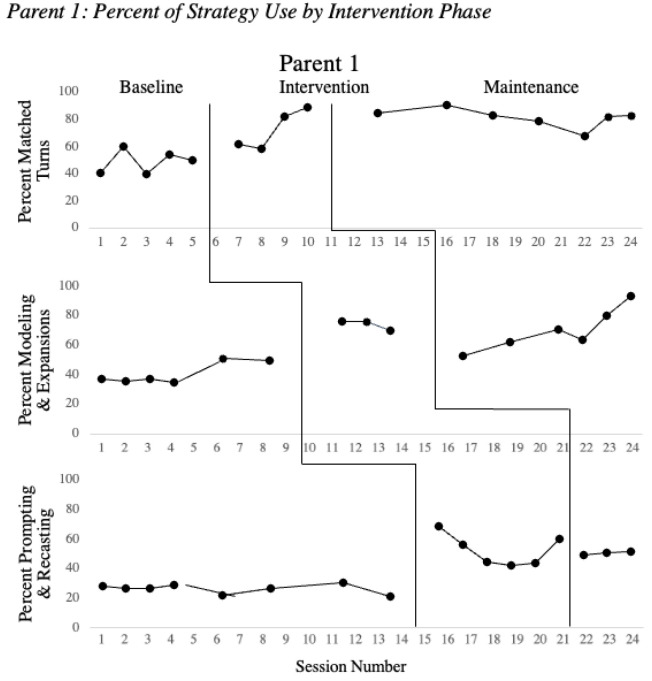
Percent of strategy use by intervention phase. Note: Percent of strategy use is presented across baseline, intervention and maintenance for the three intervention phases matched turns, modeling and expansions and prompting and recasting.

**Figure 2:**
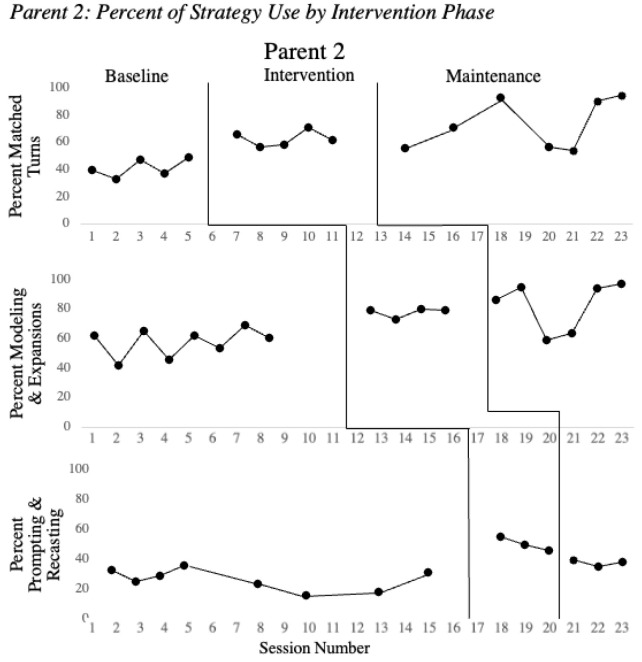
Percent of strategy use by intervention phase. Note: Percent of strategy use is presented across baseline, intervention and maintenance for the three intervention phases matched turns, modeling and expansions and prompting and recasting.

**Figure 3:**
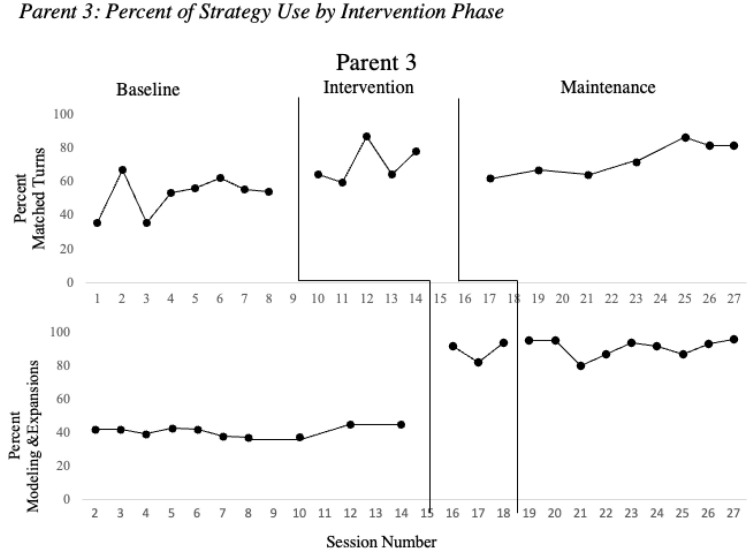
Percent of strategy use by intervention phase. Note. Percent of strategy use is presented across baseline, intervention and maintenance for the two intervention phases matched turns and modeling and expansions. Between Baseline 8 and 14, data were collected every other session for the remainder of the collected baselines.

Figure 1, Figure 2 and Figure 3 show the parent strategy use during baseline, intervention and maintenance phases for the parents of the children with clefts. All parents successfully implemented matched turns with a minimum of a 10% increase over the average of baseline performance. During the intervention phase, all three parents increased use of matched turns with coaching support by the clinicians. Once intervention for matched turns concluded, the parent’s performance remained elevated while participating in intervention sessions with a focus on new strategies.

Once a stable baseline had been established and the MVA indicated that Parents 1–3 were ready to enter intervention for modeling and expansion, all three parents increased their use of this strategy with a minimum of 10% as shown in Figure 1, Figure 2 and Figure 3. Immediately following intervention, the maintenance data demonstrated that each parent successfully incorporated these strategies in their natural play, as usage remained high without coaching from the clinician.

Based on preliminary studies of child characteristics during intervention, children needed to achieve a talking rate of at least 10 words/minute to assure sufficient opportunities for speech recasting to be effective [7]. Children 1 and 2 met these criteria and their parents were trained on prompting and recasting. Child 3 did not meet the criterion to advance to speech recasting, and intervention instead continued to focus on use of matched turns, environmental arrangement, modeling, and expansion of vocabulary. Parents 1 and 2 successfully implemented prompting and speech recasting with a minimum of a 10% increase as compared to the average of baseline values. Once Parents 1 and 2 entered the maintenance phase and direct coaching had been discontinued, their performance remained elevated compared to baseline performance, though decreased from their performance during intervention.

### 3.2. Procedural Fidelity

The parents’ use of strategies was assessed using the procedural fidelity checklist during each session until they met the fidelity criterion established for each phase of the intervention within three sessions (criterion: 75% for matched turns, 40% for modeling and expansions and 35% for prompting and speech recasting) and continued through the maintenance phase [7,8].

### 3.3. Parent Strategy Effect Sizes

Effect sizes for the intervention phases for each parent were calculated to assess intervention effectiveness. Table 3 shows the percentage of overlapping data (PND), Tau, and qualitative interpretation for the intervention phases for each mother. Throughout the course of intervention, Parents 1–3 increased the frequency with which they used each set of strategies with significant PND of *p* = 0.03 or better. Effect size as determined by Tau indicated effective and very effective findings for each intervention phase for all three mothers.

### 3.4. Parent Feedback

Following the conclusion of intervention, parents were asked to complete a satisfaction survey. Parents agreed that the instructed strategies helped them to better interact with their child. They agreed that the strategy use, and coaching components of the T-M-C-R method were useful and stated they will continue to use the provided strategies with their child following the conclusion of intervention. The parents also agreed that Zoom was a useful method of conducting intervention sessions and they were provided with useful feedback over Zoom. When asked what would be most helpful to continue the use of strategies following the end of intervention, they suggested periodic review sessions through Zoom and face-to-face meetings. They commented that the convenience of at-home Zoom sessions coupled with the parent strategies made the program especially rewarding as they were given the ability to teach their child necessary speech and language skills on their own during daily activities.

### 3.5. Child Speech and Language Characteristics

The pre–post intervention speech and language assessment results are presented in Table 4 for the three children receiving intervention. At post-intervention, all three children positively increased their consonant inventories and percentage of consonants correct. On average, PCC Total increased by 23.6% and PCC of Stops increased by 24.2% overall. Child 1 showed a 35.6% increase in PCC Total and a 30.4% increase in PCC of Stops (the focus of intervention targets) from pre-intervention to post-intervention. Child 2 demonstrated a 28.8% increase in PCC Total and a 21.1% increase in PCC of Stops from pre to post-intervention. Child 3 showed a 6.6% increase in PCC Total and a 20% increase in PCC of Stops from pre-intervention to post-intervention.

All children increased their consonant inventories from pre- to post-intervention. Child 1 also increased her word-initial consonant inventory by three consonants (∫, t∫, dʒ), and her word-final consonant inventory by nine consonants (d, k, g, θ, f, z, ∫, l, r). From pre- to post-intervention, Child 2 produced consonants in three different manner classes in the word-initial position. He increased his word-initial consonant inventory by three consonants (p, w, j) and his word-final consonant inventory by five consonants (p, t, f, n, r). At post-intervention, Child 3 produced an additional word-initial stop consonant /b/.

All three children’s speech intelligibility increased from pre-intervention to post-intervention, according to parent report on the ICS. Child 1′s intelligibility increased from an average score of 2.67 (rarely-to-sometimes understood) to an average score of 3.57 (sometimes-to-usually understood). Child 2′s average score on the ICS increased from 2.71 (rarely-to-sometimes understood) to 3.71 (sometimes-to-usually understood). Child 3′s intelligibility increased from an average score of 2 (rarely understood) to 2.29 (rarely understood).

Overall, the expressive vocabulary as reported on the CDI increased from pre-intervention to post-intervention. Child 1 showed the largest gain with her mother reporting an additional 299 total different words produced at post-intervention. Child 2′s mother reported an additional 91 words at post-intervention, and Child 3′s mother reported 20 additional words being produced at post-intervention. In addition, all children increased the number of total words and total number of different words they produced in a fifteen-minute language sample. Child 1 and 2 nearly doubled their number of different words at post-intervention while Child 3 more than quadrupled the number of different words he used from pre- to post- intervention.

**Figure 4:**
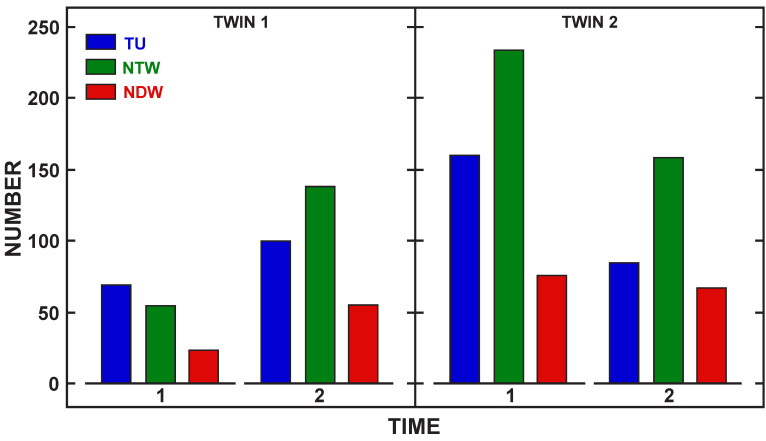
Expressive language measures comparison. Note. TU = total utterances, NTW = number of total words, NDW = number of different words. Time 1 = pre-intervention. Time 2 = post-intervention.

**Figure 5:**
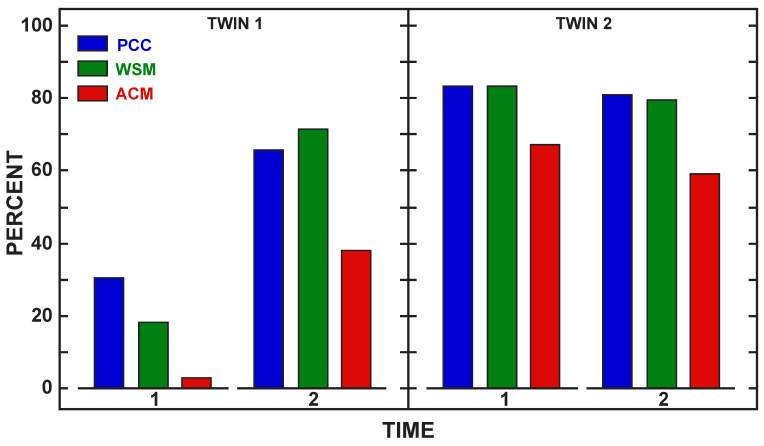
Speech measures comparison. Note. PCC = percent consonants correct, WSM = word structure match, ACM = whole word accuracy. Time 1 = pre-intervention. Time 2 = post-intervention.

While the improvement in speech and language performance pre–post was positive for all three children, it is reasonable to expect that these children would be increasing their speech and language skills over the three months of the study due to expected developmental gains for this age range. To address this issue, a comparison was made to a child without cleft palate who was a twin with Child 1. This child did not participate in the intervention sessions, but her mother was trained on the intervention strategies. Language samples were collected pre and post intervention with the twins and their mother in a naturalistic play environment for 15-min. These samples were transcribed in SALT following the sample procedures as with the other children. Table 5 shows the pre- and post-intervention assessment for the noncleft twin. Figure 4 shows the total utterances (blue bar), total words produced (green bar), and number of different words (red bar) during the language sample for Child 1 (CL/P) and her noncleft twin at pre intervention (Time 1) and post intervention (Time 2). Child 1 increased all language measures in post intervention and her twin reduced her use of language measures from pre- to post-intervention. Figure 5 shows pre–post (Time 1 and 2) intervention PEEPS results for PCC (blue bar), word structure match (WSM (green bar); percent of words with same CV structure as test targets) and whole word accuracy match (ACM (red bar): percent of words that have all consonants correct) for both twins. The data show that Child 1 made gains in all speech measures from pre- to post-intervention while her twin remained the same for the three phonological measures but talked less in the post intervention sample. These results indicate that for this pair, the child receiving intervention with the parent made gains in language and speech that approached her twin without cleft palate.

## 4. Discussion

### 4.1. Parent Use of EMT + PE Strategies

All three parents increased their strategy use substantially from baseline performance within an average of four sessions and maintained their use over nine weeks of the study. Studies of parent training using EMT strategies have shown positive results with noncleft children. Roberts and Kaiser (2015) and Roberts, Kaiser, Wolfe, Bryan and Spidalieri (2014) trained their parents of toddlers with language delays in 28 sessions over three months [14,15]. The Roberts and Kaiser studies trained their parents face-to-face, twice weekly to achieve positive results. In contrast, the present study trained parents through telepractice, three times weekly. These findings suggest that telepractice delivery of parent training can achieve a similar result as compared to face-to-face training. Additionally, the dosage of training in the present study was similar to the Roberts and Kaiser studies, but occurred over nine weeks rather than three months. Increased intensity of parent training permitted moving through the intervention phases more quickly than in Roberts and Kaiser face-to-face studies.

### 4.2. Maintenance of Strategy Use

All three parents continued strategy use for an additional three to four weeks following intervention. All parents maintained the responsive interaction, and modeling and expansion strategy use at acceptable levels. Two of the three parents were trained on prompting and speech recasting and these parents maintained the strategy use through the long-term maintenance. Recall that the third parent was not trained on prompting and recasting due to their child’s limited vocabulary. This finding is similar to the Roberts and Kaiser studies and shows that the telepractice training was effective in producing effects that were maintained for one month without intervention.

### 4.3. Child Language and Speech Outcomes

Language Outcomes: expressive vocabulary increased at post intervention for all children based on parent report (CDI) and language sample analysis. Child1 and Child 2 gained 99 and 91 words on the CDI while Child 3 gained 20 words. Post-intervention vocabulary expansion in 15-min language sample showed gains in total number of words and number of different words of an average of 59 (4–122) and 18 (7–26) words respectively, indicating that new vocabulary use was productive in communicative interaction. The two children with the fastest acquisition had larger vocabulary reported by the parents are pre-intervention while the third child had less than 10 words at pre-intervention and showed poorer receptive language performance on the PLS-5. These findings are supported by other parent implemented studies of noncleft children with speech and language delays [14,39]. These noncleft studies showed vocabulary increases between 2 and 101 words following the intervention. In a recent student by Frey et al., (2017) who studied acquisition of vocabulary in a clinician directed intervention of toddlers with and without cleft palate, they found a 90-word advantage for children receiving EMT + PE over a business-as-usual comparison group [40]. Results from the present study are well within the parameters found in other intervention studies and speak to the efficacy of our intervention delivered by parents through telepractice. Additionally, comparison of cleft-noncleft twins in this study provides evidence that the progress observed in language was not solely related to maturation as the acquisition of vocabulary in our noncleft twin remained stable during the duration of the study.

### 4.4. Speech Outcomes

Gains in consonant inventory, speech accuracy, and speech intelligibility were evident for all children with cleft in the study. However, more speech gains were observed for Child 1 and 2 who had more advanced language use at pre-intervention (CDI vocabularies above 50 words). This pattern of speech acquisition was observed in a Scherer et al. (2020) study where the children with vocabularies of at least 50 words made the most gains in speech [7]. The authors suggested that children with larger vocabularies talked more and parents were able to use the speech recasting strategy more to facilitate speech production accuracy. A recent study of an adaptation of EMT + PE in Brazil, found that significant progress in vocabulary acquisition preceded gains in speech accuracy [12]. The current study administered through telepractice showed this same pattern with Child 1 and Child 2, with vocabularies above 50 words, making greater speech gains and Child 3, with less than 50 words at pre-intervention, making the most of his gains in vocabulary with less change in speech production. The non-cleft comparison twin did not exhibit significant PCC gains, nor did she substantially increase her consonant inventory at the time of post intervention. This further suggests that parent training in EMT + PE may have a positive impact on the speech outcomes of children with CL/P beyond what would be expected from maturation alone.

### 4.5. Structural Considerations

During the assessments and intervention, speech and resonance features that could be indicative of a structural deficit were evaluated. Child 1 demonstrated mild hypernasality on vowel production but no audible nasal emission on consonant production as rated in spontaneous speech from the language sample by an SLP familiar with speech assessment for children with cleft palate. Child 1 also used compensatory articulation errors including glottal stops and pharyngeal fricatives at pre intervention but did not use pharyngeal fricatives at post intervention. Child 2 had a palatal fistula, demonstrated obligatory nasalization of voiced pressure consonants and used glottal stops at pre intervention. Despite the fistula, Child 2 did expand his consonant inventory and improve his speech accuracy. This was particularly important for this child due to a medical diagnosis delaying fistula repair. There was also suspected velopharyngeal dysfunction for Child 2 that may be occurring in addition to the palatal fistula; however, neither Child 1 nor 2 has completed standard assessment of velopharyngeal dysfunction due to age and compliance. Child 3 did not have resonance concerns or compensatory articulation errors in his speech sample; however, this child demonstrated limited spontaneous speech production and velopharyngeal function will need to be assessed further as his language increases.

## 5. Clinical Implications

Clinical practice in the field of speech pathology is currently experiencing a new growth in the area of telepractice. This study suggests that telepractice is an effective method for training parents in early intervention. Modifications of EMT + PE parent training for telepractice protocol had direct clinical implications. First, the dosage of services, (three times a week), led to rapid increases in intervention strategy use by the parents. This dosage is higher than what typically occurs in early intervention, but it allowed the parents to progress quickly through the intervention phases and suggests that this dosage improved the efficiency of delivering this intervention. Second, the use of multiple methods for assessment allows for differences in child responsiveness and home environment that could impact test administration. Methods that use parent report and natural conversational language samples between parent and child are consistently effective; however, administration of standardized language and speech naming tests required considerably more time to administer and often necessitated training of the parent so that they did not inadvertently give cues to answers. These modifications had variable results for the toddlers in this study.

## 6. Limitations

Toward the end of intervention, COVID-19 stay-at-home regulations were implemented state-wide and resulted in a disruption of daily routines for our families. All three families were adjusting to new work and home routines which is reflected in the variability in parent strategy performance toward the end of intervention. Despite this, all three parents remained actively involved in telepractice intervention sessions and all three commented that they were continually motivated by the positive changes they were observing in their children’s speech and language skills.

This study did provide a comparison to a non-cleft twin from pre–post intervention language samples with her mother and her sibling with a cleft. The language measures from the post intervention language sample revealed slightly fewer words, and rate of talking compared to the pre-intervention recording three months earlier. It should be noted that MLUm did increase from 1.71 to 2.01. Unfortunately, we did not obtain standardized assessment measures post intervention for the twin during the COVID-19 lockdown. It may be that that lack of progress in basic language sample measures could be a result of regression to the mean in her language use. However, examination of the mothers’ utterances during the language samples revealed that she was responding equally to her children’s utterances in the post sample while the pre intervention sample was dominated by the noncleft twin since her sibling was not talking much.

This study was a pilot study, and the findings should be replicated in a larger sample. In addition, speech strategies to address cleft related speech errors through telepractice should be developed and piloted to augment EMT + PE.

## 7. Conclusions

This study demonstrated that parents can successfully be trained to deliver high-quality EMT + PE intervention strategies through telepractice that was similar to parent training in face-to-face settings. Training in this approach led to positive speech and language outcomes when parents implemented strategies including environmental arrangement, matched turns, modeling and expansions, and prompting and speech recasting. All parents achieved criterion performance on the intervention strategies and maintained their use following the intervention. Additionally, all parents rated the telepractice delivery of the intervention positively but reported a preference for a hybrid of telepractice and face to face methods.

Pre-intervention and post-intervention comparison of speech and language outcomes demonstrated increased expressive vocabulary, number of different words and total words in a language sample and percent of consonants correct, percent of consonants correct of stops, consonant inventory and speech intelligibility of children with CL/P beyond what would be expected through maturation alone.

## Figures and Tables

**Table 1:** Participant Demographics.

Measure	Child 1	Child 2	Child 3	Non-Cleft Comparison
Gender	F	M	M	F
Age at Pre-Intervention Assessment	2;03	1;9	1;10	2;03
Cleft Type	Unilateral cleft lip and palate	Bilateral cleft lip and palate	Unilateral cleft lip and alveolar notch	-
Age at Lip Repair	5 months	6 months	7 months	-
Age at Palate Repair	11 months	10 months	-	-
Additional Conditions	Hemifacial microsomia	Glanzmann Thrombasthenia, palatal fistula	-	-
Hearing Status	WNL	WNL	WNL	WNL
Mother Demographics				
Age	35	33	39	35
Education Level	Some college	Bachelor’s Degree	Master’s Degree	Some college
Occupation	Retail manager	Stay at home mom	Stay at home mom	Retail manager

Note. WNL = within normal limits.

**Table 2:** EMT + PE Fidelity Rating Form.

Name:		Reviewer:				
Video Session:						
Item			0	1	2	3
Pull it all together						
The environment provides physical boundaries and contains age-appropriate toys. Environmental Arrangement						
Materials can be used to elicit multiple types of conversation and play (e.g., books, block with trucks, routines for snacks or group times). Environmental Arrangement						
The parent is responsive to the child’s interests (i.e., joins in activities, is positive and energetic, is physically accessible, is at child’s level). Matched Turns						
Time delays are used when the child requests nonverbally or with minimal verbalizations. Prompting						
Models include the child’s targets. Models, Speech Recasting						
Provide a question or a model with “say X,” if the child does not respond to the initial time delay. Models, Prompting						
Praise all correct requests, responses, and behaviors. Matched Turns						
Uses choice questions during session to elicit child targets. Prompting						
Adult language, including expansions, do not exceed 2–3 words longer than child’s MLU). Expansions						
Uses open-ended questions (i.e., “Tell me what you want?”, “What do you want to do?”). Prompting						
Expansions preserve as much of the child utterance as possible. Expansions						
The adult emphasizes the target sound in the word the child produced incorrectly when recasting. Speech Recasting						
Overall rating of EMT + PE strategies throughout video						

0: intervention strategy is not present; 1: intervention strategy needs practice; 2: intervention strategy is inconsistent; 3: intervention strategy is excellent.

**Table 3:** Intervention effect size values.

Parent	Measure	PND (%)	PND	Tau	Tau	CI 90%	Tau Qualitative Interpretation
			*p*-Value		*p*-Value		
1	Phase 1	**75**	**0.0319**	**0.9**	0.0275	0.228<>1	Effective
	Phase 2	100	0.0111	1.1333	0.0113	0.398<>1	Very effective
	Phase 3	100	0.0014	1.1	0.0026	0.499<>1	Very Effective
2	Phase 1	100	0.0025	0.84	0.0283	0.210<>1	Effective
	Phase 2	100	0.0025	0.75	0.0372	0.398<>1	Effective
	Phase 3	100	0.0111	1	0.0253	0.264<>1	Very Effective
3	Phase 1	40	0.0902	0.675	0.0283	0.113<>1	Effective
	Phase 2	100	0.0003	0.9111	0.0372	0.398<>1	Effective
Weighted Average	Phase 1	-	-	0.7979	0.0003	0.4384<>1	Effective
	Phase 2	-	-	0.9173	0	0.5532<>1	Effective
	Phase 3	-	-	1.0551	0.0003	0.5802<>1	Very effective

Note. Phase 1 = matched turns; Phase 2 = modeling and expansions; Phase 3 = prompting and recasting. Percentage of nonoverlapping data (PND) calculated based on percentage of treatment phase scores that exceed the maximum score in the baseline phase for each parent. Tau, *p* value and qualitative interpretation of the effect size based on Rakap, 2015. Weighted average for each treatment phase across parents was calculated using Tau and a qualitative interpretation provided. <>: Less than or greater than 1.

**Table 4:** Pre- and post- intervention child speech, language, and cognition data.

Measure	Assessment	Child 001		Child 002		Child 003	
		Pre-Intervention	Post-Intervention	Pre-Intervention	Post-Intervention	Pre-Intervention	Post-Intervention
PCC Total	Profiles of Early Expressive Phonological Skills (PEEPS) [35]	30.6%	66.2%	9%	37.8%	7.7%	14.3%
PCC Stops		44%	74.4%	9.3%	31.4%	20%	40%
Consonant inventory in the initial position of words		p, b, t, d, k, g, f, m, n, l, w, h, j, ʔ, ʕ	p, b, t, d, k, g, f, **∫**, **t∫**, **dʒ**, m, n l, w, h, ʔ	b, k, m, n, ʔ	**p**, b, m, n, **w**, ʔ, $\overset{\sim }{\underset{\circ }{m}}$, **j**	0	**b**, ***d***, ***h***
Consonant inventory in the final position of words WSM ACM		p, t, s, n, ŋ, ʔ 18.6% 3.4%	p, t, **d**, **k**, **g**, θ, **f**, **s**, **z**, **∫**, n, ŋ, **l**, **r** 71.7% 38.3%	k, l, ʔ, $\overset{\sim }{\underset{\circ }{m}}$ 9.3% 0%	**p**, **t**, k, **f**, **n**, **l**, **r**, ʔ, $\overset{\sim }{\underset{\circ }{m}}$ 37.8% 6.7%	t, *h* 0% 0%	***p***, *t*, *h* 0% 0%
Expressive Communication Standard Score	Preschool Language Scales (PLS-5) [34]	88	103	94	-	88	-
Auditory Comprehension Standard Score		106	104	117	-	88	-
ICS Average Score	Intelligibility in Context Scale (ICS) [36]	2.67 Rarely-to-sometimes	3.57 Sometimes-to-usually	2.71 Rarely-to-sometimes	3.71 Sometimes-to-usually	2 Rarely	2.29 Rarely
MCDI Words Produced	McArthur Bates Communicative Development Inventory (CDI) [33]	233	532	70	161	2	22
Percentile		15	40	35	30–35	<5	<5
MCDI Word Forms		1/25	11/25	0/25	1/25	0/25	0/25
Percentile		5–10	15–20	55	30	35	10
MCDI Complexity		2/37	22/37	0/37	0/37	0/37	0/37
Percentile		20	69	70	30	55	30
NDW	Language Sample	37	63	14	34	2	9
MLUm		1.26	2.07	1.38	1.10	1.19	1.15
TNW		86	208	36	88	19	23
WPM		8.67	21.82	4.43	7.37	5.3	7.36
Cognitive Scaled Score (Pre-test only)	Bayley Scales of Infant Development—III [32]	10	-	10	-	7	-
Cognitive Percentile Score		50%	-	50%	-	16%	-

Note. PCC Total = total percent consonants correct. PCC Stops = percent consonants correct of stops. WSM = word structure match. ACM = whole word accuracy. NDW = Number of Different Words. MLUm = Mean Length of Utterance in Morphemes. TNW = Total Number of Words. WPM = Words per Minute; Consonants added at post-intervention are bold. Consonants presented in italics were observed during language assessments. Parents were instructed on how to complete the MCDI at both timepoints.

**Table 5:** Pre- and post- intervention child speech, language, and cognition data.

Measure	Assessment	Non-Cleft Comparison	
		Pre-Intervention	Post-Intervention
PCC Total	Profiles of Early Expressive Phonological Skills (PEEPS, Stoel-Gammon, 2013) [35]	83.6%	81.2%
PCC Stops		95.8%	84.5%
Consonant Inventory in the Initial Position of Words		p, b, m, w, f, t, d, s, n, k, g, h, ∫	p, b, m, w, f, t, d, s, **z**, n, **l**, k, g, h, **θ**
Consonant Inventory in the Final Position of Words WSM ACM		p, m, f, θ, t, d, s, z, n, l, t∫, k, g 83.6% 67.3%	p, **b**, m, f, t, d, s, n, l, k, g, t∫, **ŋ** 79.7% 59.3%
Expressive Communication Standard Score	Preschool Language Scales (PLS-5, Zimmerman et al., 2011) [34]	110	-
Auditory Comprehension Standard Score		124	-
ICS Average Score	Intelligibility in Context Scale (ICS, McLeod, Harrison, and McCormack, 2012a) [36]	4.17 Usually	- -
MCDI Words Produced	McArthur Bates Communicative Development Inventory (CDI, Fenson et al., 2007) [33]	413	-
Percentile		35–40	-
MCDI Word Forms		14/25	-
Percentile		70	-
MCDI Complexity		22/37	-
Percentile		55–60	-
NDW	Language Sample	76.5	68
MLUm		1.71	2.01
TNW		234	160
WPM		15.50	10.68
Cognitive Scaled Score (Pre-Test Only)	Bayley Scales of Infant Development—III (Bayley, 2006) [32]	9	
Cognitive Percentile Score		37	

Note. NDW = number of different words; MLUm = mean length of utterance in morphemes; TNW = total number of words; WPM = words per minute. Consonants added at post-intervention are bold.

## Data Availability

The data presented in this study are available in the present article.

## Supplementary Materials

The following are available online at https://www.mdpi.com/article/10.3390/children8090736/s1.
