# Supplementary material for: Enhanced Milieu Teaching with Phonological Emphasis: A Pilot Telepractice Parent Training Study for Toddlers with Clefts
Source: Children (Basel). 2021 Aug 26;8(9):736. doi: 10.3390/children8090736 (PMC8471217; doi:10.3390/children8090736)
Supplement: Supplementary file 1 [file children-08-00736-s001.zip › children-1325031-supplementary.pdf]

Supplementary Materials

Definition of Intervention Strategies

1. *Environmental Arrangement*
    a. Big: arrangement of the outdoor or indoor equipment or furniture to facilitate engagement and manage access to materials e.g., toys out of reach but visible.
    b. Little: strategies to maintain engagement and facilitate requesting during the interaction with the child
        i. Limited portions
        ii. Broken items
        iii. Choices
        iv. Sabotage
        v. Missing pieces
2. *Responsive Interaction: Matched turns*
    a. Definition: active engagement during play from the parent as any nonverbal or verbal act that the child makes is "matched" with a parent response [8].
    b. Key principles:
        i. Follow the child's lead and join in on activities the child showed interest in.
        ii. Parents were encouraged to step back and allow the child to choose the materials he/she would like to engage with.
        iii. Playing face-to-face was emphasized with this strategy to promote social engagement and communication.
        iv. Parents were instructed to show the child a toy rather than telling the child what a toy was
            1. For example, parents were asked to pick up a toy, show it to the child and allow multiple seconds of wait time to see if the child would vocalize about the toy before the parent labeled it.
3. *Modeling and Expansions*
    a. Definition: Modeling and expansions are used to encourage language through the parent demonstrating and showing (pointing) to support language and expanding on words the child did produce [8].
    b. Key Principles:
        i. Parents were trained to model words by labeling an object, labeling the result of an action, labeling a change in location, and/or labeling an action he/she performs. For example, a parent might provide a model by opening a box of cars, giving one to the child, and verbally modeling the word "car".
        ii. Parents were taught to expand on the utterances that the child was able to produce.

iii. Add on to the child's utterance using a new or different word when the child produced a spontaneous utterance, or when the child imitates the parent's utterance.
iv. Turn the child's word into a sentence. For example, if a child were to push a car down a ramp and say "down", the parent could expand on this utterance by responding with "the car went down".

4. *Prompting and Speech Recasting*
   a. Definition: Prompting and speech recasting are used to give the child the opportunity to use new words and give the parent an opportunity to model the correct production of phonemes [8].
   b. Key Principles:
      i. Parents were instructed to direct their child to say a word by providing them with appropriate support when the child makes a nonverbal request, uses an incorrect word, and/or uses a general word such as "this" or "that".
      ii. The hierarchy of increasing support included asking an open question, giving a choice, or asking their child to say a specific word.
      iii. An open question provides the least amount of support and is used when the child already knows the word for what he/she is requesting. For example, if the child is playing with toy animals the parent might say "what are the animals doing?".
      iv. Giving a choice provides a child with a mid-level of support and includes giving the child a verbal model of the word he/she is intending to request.
      v. Parents were trained to hold up two objects without saying anything and wait for the child to indicate which one he/she wants.
      vi. If the child did not respond verbally, the parent was told to give the child a verbal choice. For example, if the child was playing with a baby doll, the parent held up two bottles, and the child said "milk," the parent would then hand the child the bottle of milk. If the child did not say "milk," but instead pointed to the bottle full of milk, the parent would say, "milk or juice" and then wait for the child to respond verbally.
      vii. Using a "say" prompt gives the child the highest level of support as parents explicitly prompt their child to "say _____". "Say" prompts are especially useful in situations in which the word is one that the child does not know or does not say often.
      viii. Speech Recasting: Parents were trained to repeat their child's word immediately following an incorrect production, emphasizing the correct sound. For example, if the child said "gall" for "ball" the parent was instructed to respond with "ball," while emphasizing the /b/ sound with proper articulatory placement.
